# The role of noninvasive ventilation in patients with “do not intubate” order in the emergency setting

**DOI:** 10.1186/2197-425X-3-S1-A172

**Published:** 2015-10-01

**Authors:** M Vilaça, C Dias, I Aragão, G Campello

**Affiliations:** Medicine Integrated Master (MIM), Instituto de Ciência Biomédicas Abel Salazar, Porto, Portugal; Information Sciences and Decision on Health Department (CIDES), Faculdade de Medicina da Universidade do Porto, Porto, Portugal; Intensive Care Unit (UCIP)- Centro Hospitalar do Porto, Porto, Portugal

## Introduction

Noninvasive ventilation (NIV) has been increasingly used in patients with a “do not intubate” order (DNI). In this group of patients the use of NIV in intensive care units was associated with no decrease in health-related quality of life (HRQOL). Its impact on the clinical and HRQOL in the emergency setting is still unknown as is its utility in symptom relief in the end-of-life care.

## Objectives

To evaluate the impact of the use of NIV in the outcome and HRQOL in patients with DNI order admitted to the emergency room.

## Methods

Prospective cohort study of all patients who receive NIV for acute or acute-on-chronic respiratory failure with DNI order admitted to the emergency room of a tertiary care, university-affiliated, 600-bed hospital between January and December 2014. Patients were divided in two groups: those who had a DNI order in the context of withhold therapy decision and those in whom all treatment, including NIV was provided for symptom relief. For HRQOL evaluation SF-12 was used. This evaluation was made only in the first group of patients. Long-term outcome was evaluated at 90 days after hospital discharge by a telephone interview.

## Results

During the study period 1727 patients were admitted to the emergency room, 243 were included in the study and 70 (29%) had a “do-not-intubate order”, of those 29 (41%) received NIV for symptom relief. The median age was 82 years in the first group and 79 years in the second (p = 0,299). Active cancer [7% (n = 10) vs 35% (n = 3), p = 0,004] and neuromuscular diseases [0% vs. 17% (n = 5), p = 0,010] were more prevalent in the group undergoing symptom relief treatment. NIV was stopped in 20% (n = 8) of patients in the first group and in 59% (n = 17) in the second group, due to lack of clinical benefit (p < 0.001). The hospital mortality rate was 37% (n = 15) in the first group and 86% (n = 25) in the symptom relief group (p < 0,001). Among the group of patients discharged from hospital, 24% (n = 6) of the first group and all patients from the second group were dead at the point long-term outcome evaluation. No significant decline in HRQOL was observed at 90 days when compared to baseline.

## Conclusions

A DNI order was present in 29% of patients who received NIV for acute or acute-on-chronic respiratory failure in the emergency room. Long-term outcome translated to a 49% survival rate in the group of patients with a DNI order in whom NIV was used as a treatment with no decrease in HRQOL compared to baseline. NIV did not provide significant symptom relief in more than half of the patients in whom it was used for that purpose.

**Figure 1:**
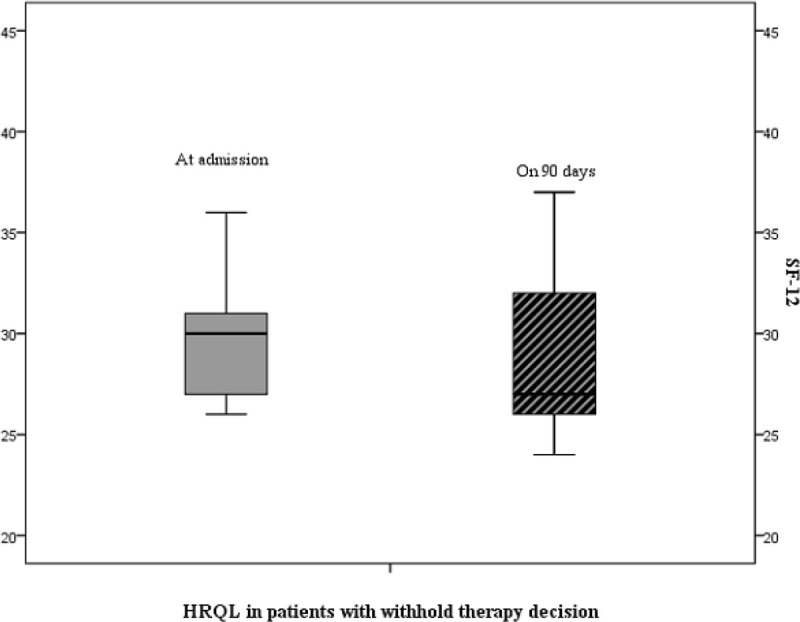
**HRQOL in patients with withhold therapy decision.**
